# Mast cells are essential intermediaries in regulating IL-33/ST2 signaling for an immune network favorable to mucosal healing in experimentally inflamed colons

**DOI:** 10.1038/s41419-018-1223-4

**Published:** 2018-12-05

**Authors:** Zhigang He, Jian Song, Jie Hua, Muqing Yang, Yuanyuan Ma, Tianyu Yu, Junlan Feng, Bin Liu, Xiaodong Wang, Yue Li, Jiyu Li

**Affiliations:** 1 Department of General Surgery, Shanghai Tenth People’s Hospital, School of Medicine, Tongji University, No. 301 Middle Yan’ Chang Road, 200072 Shanghai, P. R. China; 2 Department of Pancreatic Surgery, Fudan University Shanghai Cancer Center, No. 270 Dong’ An Road, Shanghai, P. R. China; 3 Department of Obstetrics and Gynecology, Shanghai Tenth People’s Hospital School of Medicine, Tongji University, 301 Middle Yanchang Road, 200 072 Shanghai, P. R. China

## Abstract

Mast cells (MCs) are potent tissue-resident immune cells that are distributed in the intraepithelial space of the intestine and have been implicated in regulating immune homeostasis and coordinating epithelial responses in inflamed mucosa of inflammatory bowel disease (IBD). IL-33 functions as an endogenous danger signal or alarmin in inflamed intestine segments. MCs highly express the IL-33 receptor ST2. However, the mechanisms underlying the immune regulation of MC-dependent IL-33/ST2 signaling at the barrier surface of the intestine remain largely unknown. We confirmed that MCs are required for the effective resolution of tissue damage using an experimental colitis model that allows for conditional ablation of MCs. After elucidating the IL-33 signaling involved in MC activity in the context of intestinal inflammation, we found that the function of restricted IL-33/ST2 signaling by MCs was consistent with an MC deficiency in response to the breakdown of the epithelial barrier. We observed that a tissue environment with a spectrum of protective cytokines was orchestrated by MC-dependent IL-33/ST2 signaling. Given the significant downregulation of IL-22 and IL-13 due to the loss of MC-dependent IL-33/ST2 signaling and their protective functions in inflammation settings, induction of IL-22 and IL-13 may be responsible for an immune network favorable to mucosal repair. Collectively, our data showed an important feedback loop in which cytokine cues from damaged epithelia activate MCs to regulate tissue environments essential for MC-dependent restoration of epithelial barrier function and maintenance of tissue homeostasis.

## Introduction

To function properly, the intestine immune system detects a wide variety of agents over time, maintaining tolerance of commensal microbiota and dietary antigens while retaining the ability to launch an effective immune response against invading pathogens.^1^ Disorders of the immune system manifest as inflammatory bowel disease (IBD), in which continual activation of the mucosal immune response in the gastrointestinal tract leads to chronic remittent and progressive inflammation.^2,3^ Colitis appears to be involved in chronic engagement of the stress response of epithelial barrier and dysregulation of a carefully balanced immune system, which represents the underlying pathogenesis.^4,5^ Studies implicate crosstalk between the epithelial barrier and the mammalian immune system in which damaged epithelial cells release cytokine signals that activate sentinel immune cell populations that may result in mucosal healing with epithelial regeneration and immune network regulation.^6–9^ Understanding the details of regulatory mechanisms of the intestinal immune system that direct tissue protection and remodeling is key to identifying new therapeutic targets for IBD.

Mast cells (MCs) are tissue-resident immune cells that are preferentially distributed throughout barrier tissues such as the skin and mucosa, including the intestinal intraepithelial space.^10^ MCs participate in several physiological processes and are important in initiation and regulation of immune reactions to environmental stimuli that occur in their homing tissues. The functions of MCs during colitis development has drawn substantial interest in recent years.^11–13^ Although some evidence exists on a pathogenic role for intestinal MC activation in experimental colitis, conclusive data associated with colon inflammation are lacking.^14,15^ An article on *immunity* found that factors weakening Treg cell function most likely prevail during the acute phase. However, when inflammation subsides, MCs may have more prominent functions, preventing excessive tissue damage and development of chronic inflammation.^16^ This hypothesis has been confirmed by evidence.^12,13,17^ The hallmarks of IBD are a dysregulated intestinal immune response in which MCs accumulate in the inflamed gut of IBD patients. However, the specific functions of MCs in IBD development remain to be elucidated, especially the aberrant interactions between MCs and epithelial cells or other underlying resident cells, which are fundamental for immunopathological regulation.

The importance of events involved in stress-triggered inflammation and tissue remodeling at mucosal tissues in orchestrating immune response is highlighted by the effects of tissue-derived cytokine signals in development of the underlying immune network of IBD. Studies have advanced considerable interest in interleukin-33 (IL-33) as a central instigator and candidate for therapeutic intervention of colitis.^18–20^ IL-33 is released as one of the earliest signaling molecules following epithelial damage or stress. Abundant IL-33 receptors (ST2) are expressed on MCs and coordinate spatially and temporally with IL-33 signaling, which may trigger a key regulatory amplification loop involved in immune homeostasis.^13,21,22^ As key effector cells to IL-33 signaling, MCs are recognized as driving production of high amounts of the (T helper) Th2 cytokines IL-5, IL-9, and IL-13.^23,24^ In colitis, the interaction of MCs with epithelial cells depends on the inflammatory stage and activation of the tissue repair program. However, how the IL-33/ST2-dependent MC response contributes to IBD development remains largely unknown.

We previously showed that MCs participate in the tight regulation of multiple immune responses.^25–27^ Our interest in MC function and adaptation to the inflammatory environment developed with our investigation of the infiltration of intestinal mucosal MCs in the remission phase of dextran sodium sulfate (DSS)-induced colitis. Based on studies that implicate MCs in the IL-33/ST2 axis, we investigated and characterized the unknown function of MCs in this setting when perturbed in IBD tissue environments. We confirmed that MCs are required for the effective resolution of DSS-induced colitis and are involved in the tissue environment that favors mucosal healing. In addition, this beneficial effect from MCs seemed to be facilitated by IL-33/ST2 signaling that increased the release of IL-13 and IL-22.

## Results

### MC infiltration increased during the DSS-induced colitis remission phase

**Fig. 1:**
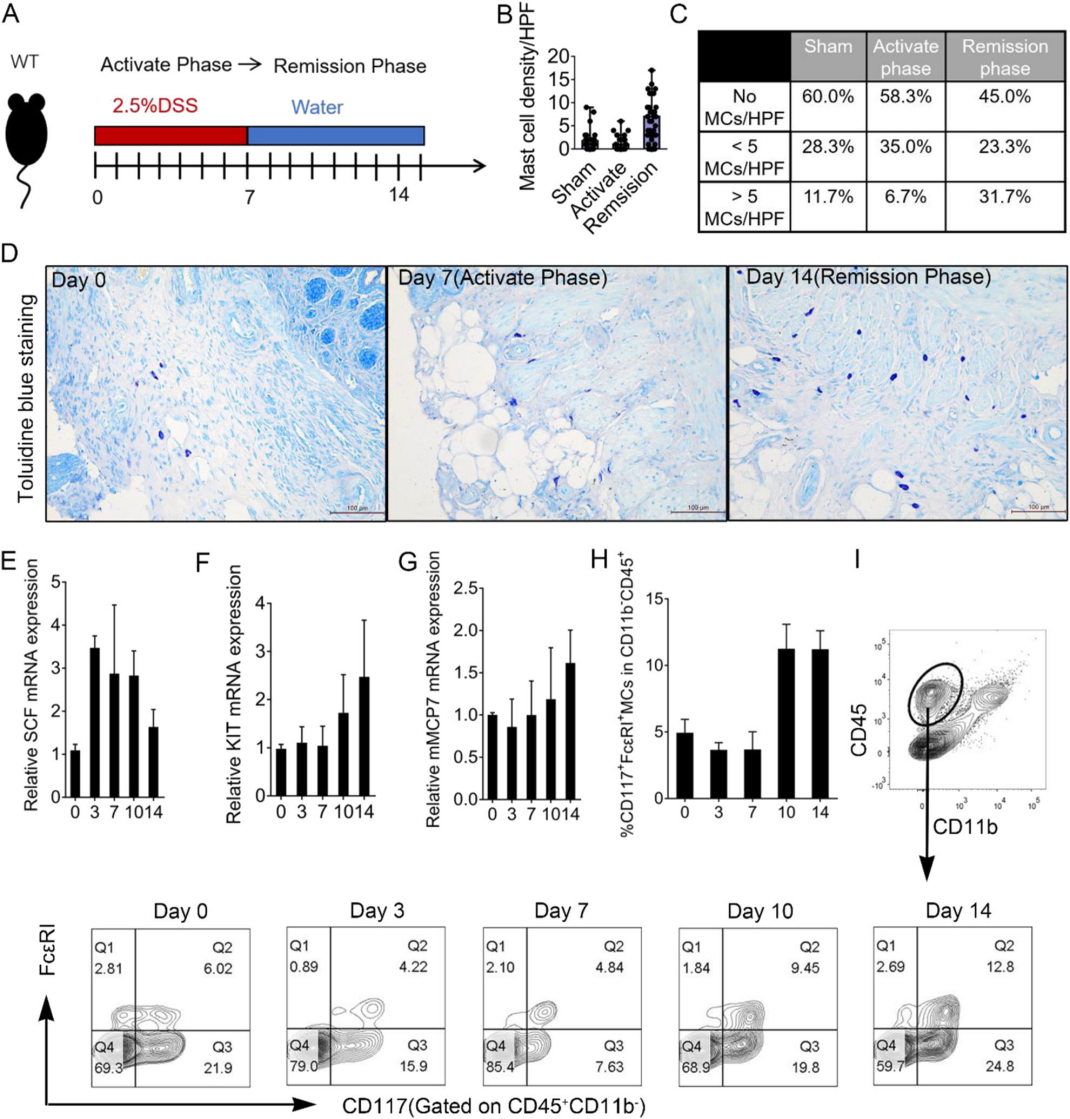
MC infiltration in colon during colitis development. **a** Experimental scheme of DSS administration. **b**, **c** Number and density of MCs in colon tissue section (*n* = 60 from 6 mice/group). **d** Representative toluidine blue stain of colon sections at Day 0, Day 7 (activate phase), and Day 14 (remission phase). mRNA expression for **e** SCF, **f** KIT, and **g** MCP-7 from real-time quantitative PCR. Data were pooled from three repeated experiments. **h**, **i** MC percentage in LP infiltrating cells analyzed by flow cytometry. Data were pooled from three different experiments. Data are presented as mean ± SD

Colitis was induced with 2.5% DSS in drinking water for 7 days. The activating phase was from day 3 to day 7 and the remission phase from day 10 to day 14 after DSS withdrawal (Fig. 1a). Histology change of colon by H&E staining during DSS-induced colitis was showed in Figure S1. We found MC numbers dramatically increased during the remission phase (Fig. 1b). The proportion without MCs was as high as 60% under basal conditions (Fig. 1c), and distributed throughout the outer layers of the muscularis propria at day 0. As colitis progressed, MCs tended to move from the outer to the inner intestinal layers, closer to the sites of mucosal regeneration (Fig. 1d). We found SCF mRNA increased in tissues mainly at days 3, 7, and 10 after DSS administration compared with sham-operated mice (Fig. 1e). In addition, expression level of c-KIT and mMCP7 increased only during remission phase (Fig. 1f, g). These results suggested that MCs develop in an SCF-dependent manner in a pathological process before mucosal repair. The percentage of FcεRIα + CD117 + MCs among infiltrating cells during the activation phase from day 3 to 7 was similar to the percentage at day 0. During the remission phase after DSS withdrawal (from 10 to day 14), we detected more than two-fold more infiltrating MCs (Fig. 1h, i).

### MC deficiency in KIT ^Wsh^ mice results in tissue repair program destruction in colitis models

**Fig. 2:**
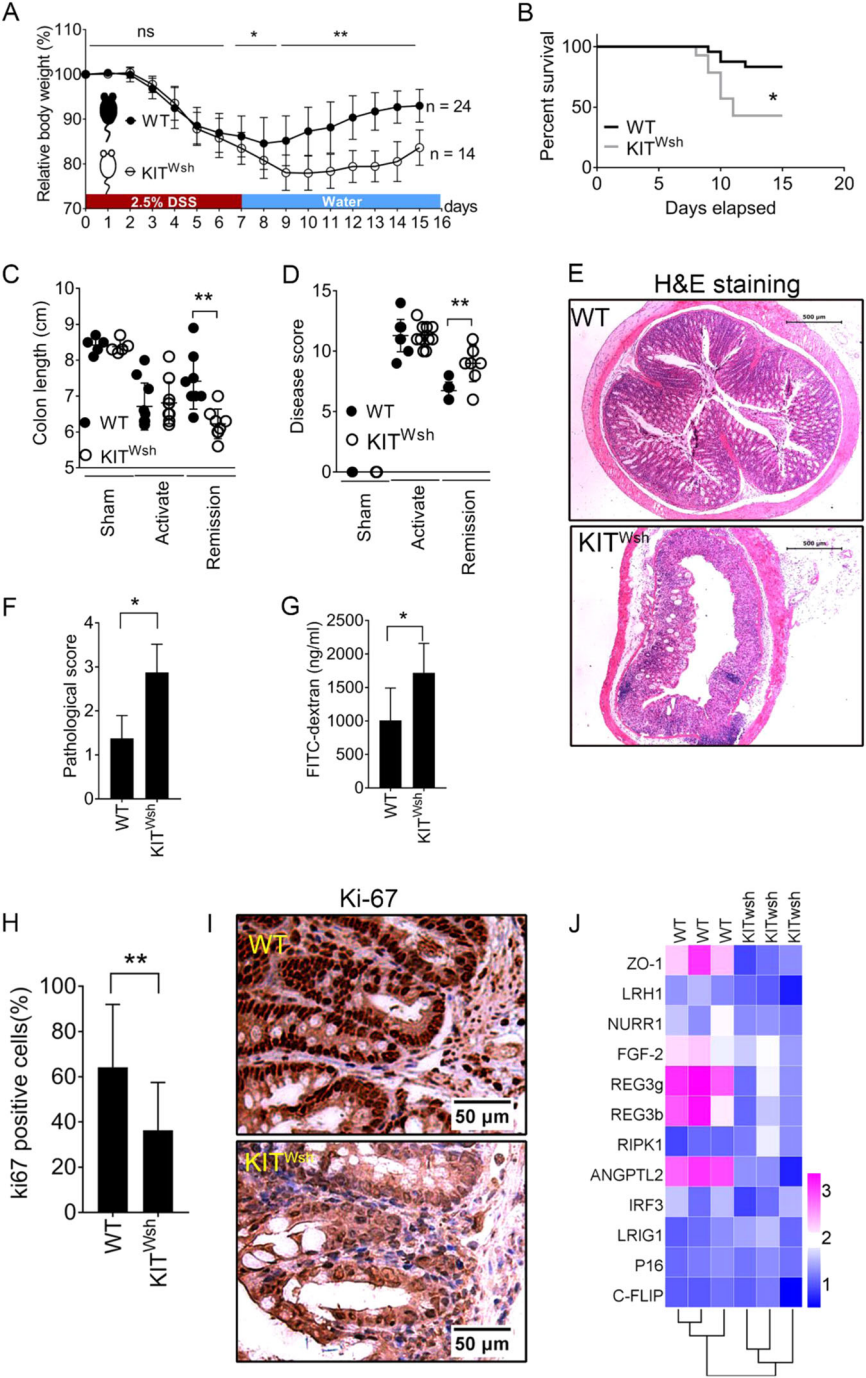
KIT ^Wsh^ mice fail in timely recovery from colitis due to MC deficiency. **a** Alterations in body weight as the percentage of initial weight at the start of experiments during a 15-day observation (24 WT mice and 14 KIT ^Wsh^ mice). **b** Survival rate for WT and KIT ^Wsh^ mice after DSS insult. **c** Whole colon lengths from sham, activation phase, and remission phase. **d** Disease score evaluated as weight loss, feces, rectal bleeding, and general appearance as indicated. **e** Representative H&E staining images of transverse colon. **f** Pathological scores from colonic sections, calculated as indicated. **g** Serum FITC-dextran quantified as a measure of intestinal permeability. **h** Percentage of positive cells for Ki-67 immunostaining. **i** Representative stain of Ki-67. **j** Heat map of relative transcriptional level of selected mRNA involved in mucosal recovery and homeostasis maintenance. Data are mean ± SD. Student *t-*test, *P < 0.05; **P < 0.01

DSS administration caused significant body weight loss with a similar progression in WT and KIT ^Wsh^ mice (Fig. 2a). In the activation phase, there are no differences in survival rate, colon length, or disease score between the WT and KIT ^Wsh^ groups (Fig. 2b–d). MC deficiency mainly affect the colitis recovery (Fig. 2a). Survival rates were also significantly decreased in KIT ^Wsh^ mice during a 15-day observation and deaths occurred mainly around the days of DSS withdrawal (Fig. 2b). An analysis of intestines in the remission phase showed that KIT ^Wsh^ mice has delayed resolution of inflammation and impaired repair of damaged mucosal tissue, which led to a continuing severe colon inflammation condition characterized by shorter colon length and higher disease scores and pathological evaluations (Fig. 2c–f). Intestinal permeability was examined using FITC-labeled dextran assays. We found that KIT ^Wsh^ mice had significantly high intestinal permeability even in the recovery phase (Fig. 2g).

Expression of Ki67 in both WT and KIT ^Wsh^ mice was significantly higher in WT mice and was associated with a better recovery (Fig. 2h, i). Expression of zonula occludens-1 (ZO-1) was decreased in tissue from KIT ^Wsh^ mice compared to WT mice, suggesting that MCs may benefit tight junctions and signal transduction between epithelial cells. The expression of basic fibroblast growth factor (FGF-2, bFGF, or FGF-β) and angiopoietin-related protein 2 (ANGPTL2) was significantly lower in KIT ^Wsh^ tissue. They are important for maintaining tissue homeostasis and promoting tissue repair. MC deficiency in KIT ^Wsh^ mice also influenced expression of the innate immune genes Reg3γ and Reg3β, which were expressed by diverging epithelial cell types and important for defense peptides in regulating intestinal inflammation^28^ (Fig. 2j). These diverse effects were largely consistent with the function of intestinal MCs. Although we did not detect differences between WT and KIT ^Wsh^ mice in the remaining tested genes associated with tissue protection and intestine homeostasis, the results suggested a limited range of meaningful effects from MCs.

### BMMC-reconstituted KIT ^Wsh^ mice exhibit a tissue repair activity in DSS-induced colitis similar to the WT counterpart

**Fig. 3:**
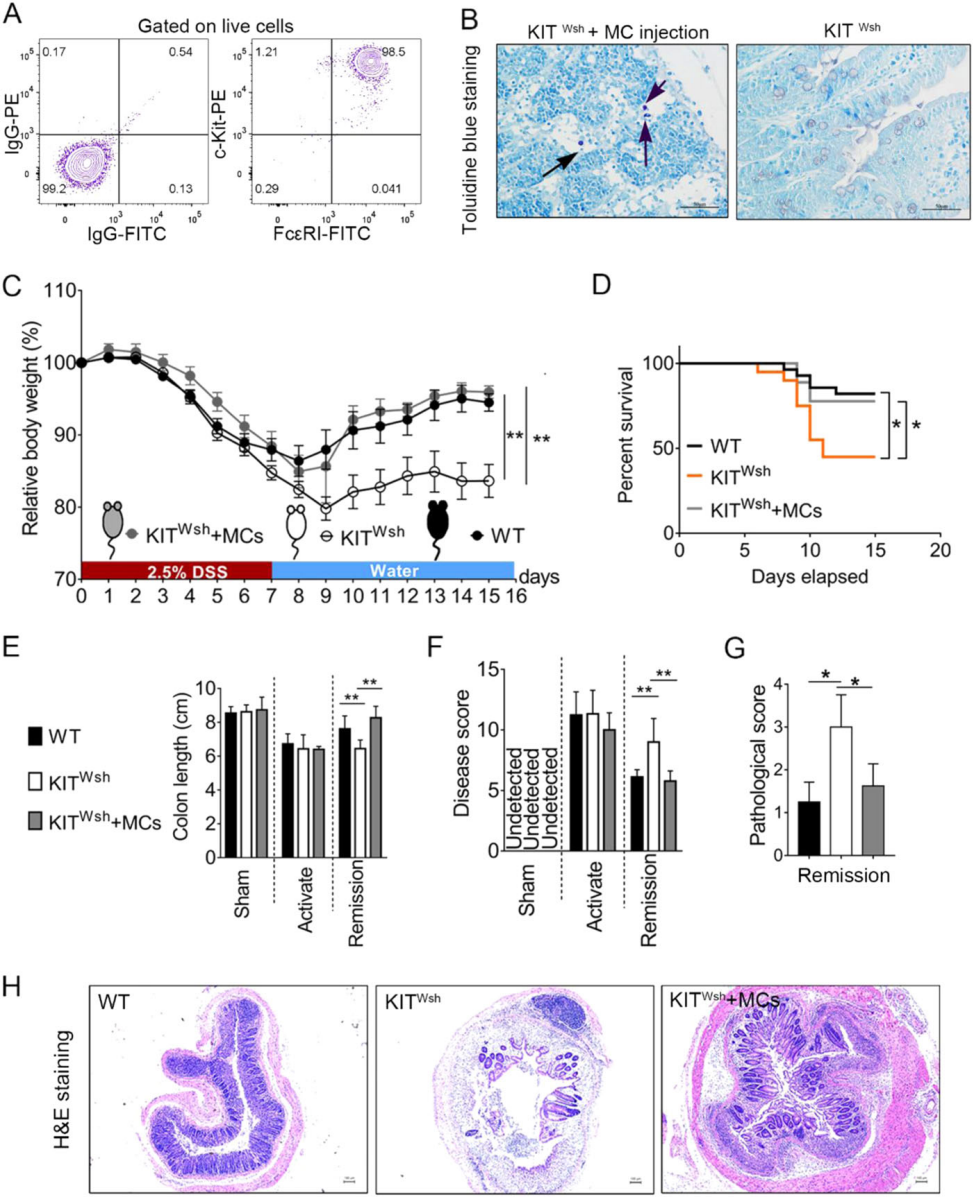
Comparison during DSS-induced colitis between WT, KIT ^Wsh^, and KIT ^Wsh^ reconstituted mice. **a**Flow cytometric evaluation of the purity of BMMC preparation. Three different experiments were repeated. **b** Identification of MC presence in the colon tissue of reconstituted KIT ^Wsh^ mice by using toluidine blue staining (8 KIT ^Wsh^ mice were used). **c** Percent difference of body mass from day 0 between different groups (KIT ^Wsh^  +  MCs mice: *n* = 8; KIT ^Wsh^ mice: *n* = 24; WT mice: *n* = 31). **d** Survival rate of WT and KIT ^Wsh^, and reconstituted KIT ^Wsh^ mice after DSS insult. **e** Whole colon lengths from sham, activation phase, and remission phase. **f** Disease score evaluated as weight loss, feces, rectal bleeding, and general appearance as indicated. **g** Pathological scores from colonic sections, calculated as indicated. **h** Representative H&E staining images of transverse colon at day 14. Data are mean ± SD. Student *t-*test, *P < 0.05; **P < 0.01

To further confirm that MCs are required for the effective resolution of DSS-induced colitis in mice, i.p. injections of BMMCs into KIT ^Wsh^ mice (Kit ^Wsh ^+ MCs) have been used in this study. The purity of MCs, evaluated as a percentage of c-Kit+ and FcεRIα+ cells, was >95% (Fig. 3a). After 5 weeks of MC injections, we detected the MC populations in the gut tissue (Fig. 3b). Body mass change in reconstituted mice was consistent with that of the WT counterpart during colitis, whereas impaired recovery occurred after the activate phase in Kit ^Wsh^ mice, suggesting that MCs are required for the effective resolution of DSS-induced inflammation (Fig. 3c). In addition, reconstituted mice obtain a similar survival rate as that of the WT (Fig. 3d). Corresponding to the above results, we detected changes in intestinal length and disease score similar to WT in reconstituted KIT ^Wsh^ mice (Fig. 3e, f). During the remission phase of colitis, the crypt architecture and pathological score of reconstituted mice was more similar to WT than to KIT ^Wsh^ mice at the same time point (Fig. 3g, h).

### IL-33/ST2 signaling is involved in MC activity during colitis

**Fig. 4:**
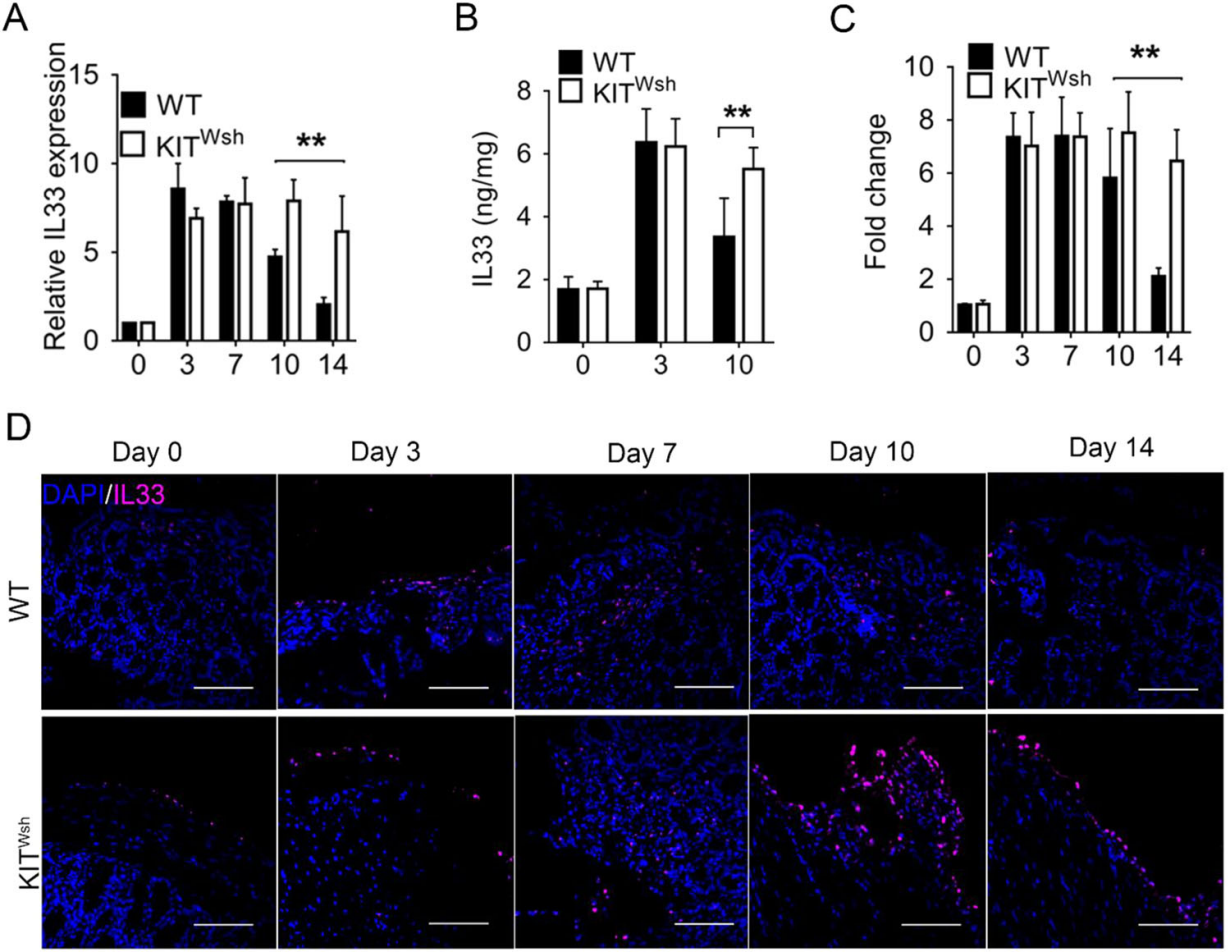
Levels of IL-33 during both the activate and recovery phases of DSS-induced colitis. **a** Relative mouse IL-33 mRNA expression in DSS-induced colitis. Data were pooled from three different experiments. **b** IL-33 protein detected by ELISA at days 0, 3, and 10 (*n* = 6/group). **c** Analysis of immunofluorescence for IL-33. **d** Representative images of IL-33 immunostaining in colonic epithelium. Data are mean ± SD. Student *t-*test, *P < 0.05; **P < 0.01

**Fig. 5:**
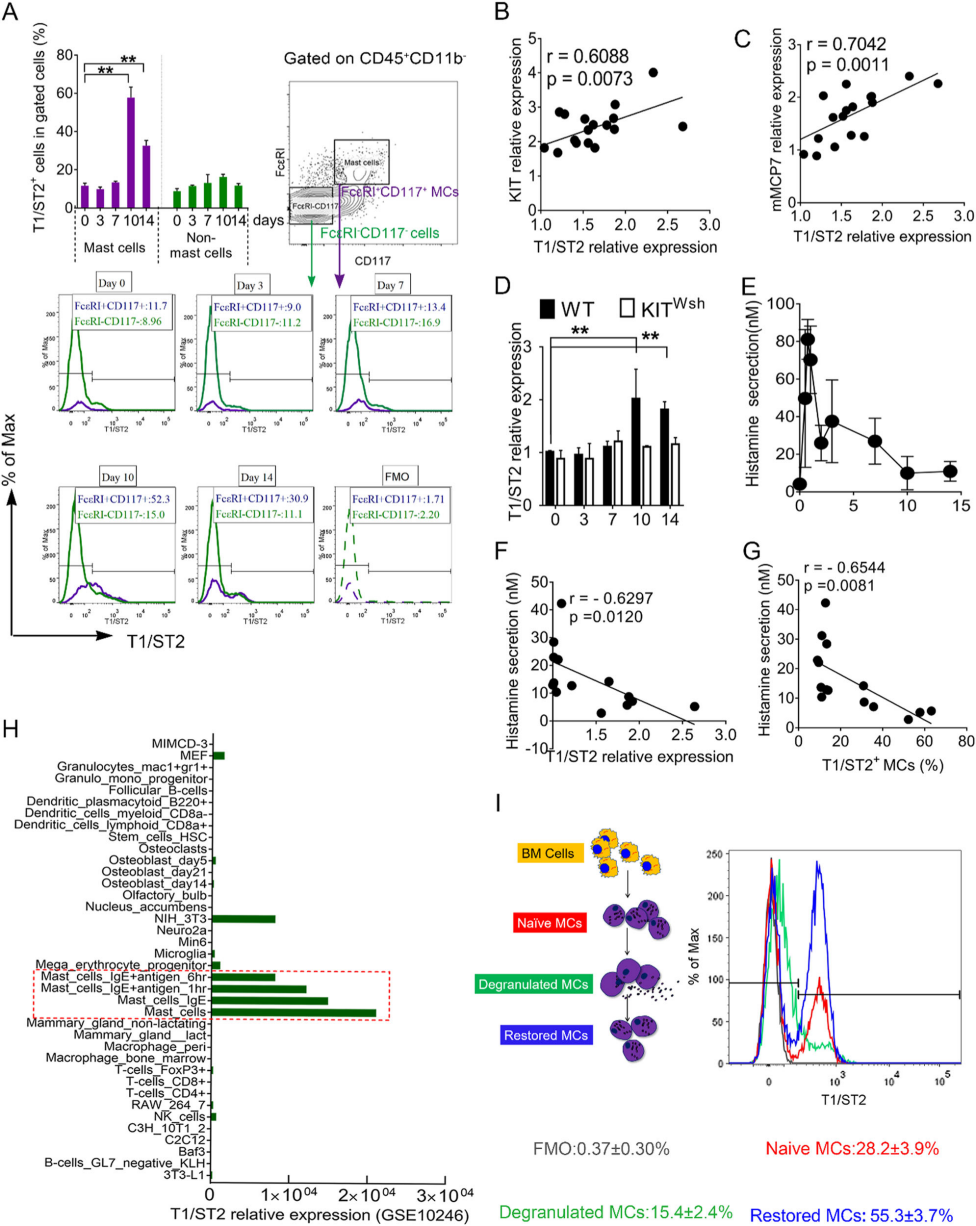
Change of T1/ST2 expression in MCs during colitis **a** Mean percentage and representative plots of T1/ST2(IL33R) for LP-infiltrating MCs and non-MCs. Correlation between T1/ST2 expression and **b** KIT expression, **c** MCP-7 expression. **d** T1/ST2 mRNA expression during colitis. **e** Histamine concentration in sera. **f** Correlation between T1/ST2 relative expression and histamine secretion. **g** Correlation between T1/ST2^+^ MCs percentage and histamine secretion. **h** Analysis of T1/ST2 relative expression in different cell types. **i** Mean percentage and representative plots of T1/ST2(IL33R) expression on naïve, degranulation (De), and degranulation-recovery (Re) MC populations. Data are mean ± SD. Student *t-*test, *P < 0.05; **P < 0.01

Similar to previous research on human IBD^29^ and mouse models,^30^ we found that mRNA/protein level of IL-33 was significantly elevated in inflamed colon (Fig. 4a, b). Immunostaining for IL-33 further confirmed these findings (Fig. 4c, d). IL-33 expression in WT mice decreased in the remission phase at days 10 and 14, but not in KIT ^Wsh^ mice (Fig. 4a–d). The percentage of T1/ST2 + MCs increased significantly at days 10 and 14 compared to day 0. Some immune cells including ILC2s, Th2 cells, and Treg cells may also express T1/ST2. However, we detected only a slightly increase in ST2 expression from non-MCs (Fig. 5a). Significant positive correlations were seen between T1/ST2 expression and expression of MC-specific markers Kit and mMCP7 (Fig. 5b, c). We detected significantly increased T1/ST2 expression in WT but not KIT ^Wsh^ mice during the remission phase of DSS-induced colitis (Fig. 5d). We focused on whether MC degranulation affected T1/ST2 expression. In colitis development, MCs appeared to degranulate in the activation phase and primarily in the first 2 days (Fig. 5e). In addition, MC degranulation in the colon primarily occurred at the early stage of colitis (Figure S2a, b). MC degranulation was also negatively correlated with T1/ST2 expression in MCs (Fig. 5f, g). Consistent with the results from the colitis model, degranulation contributed to decreased T1/ST2 expression in the activated cell population compared to the naïve MC population (Fig. 5i). By reviewing and analyzing data obtained from a database (GSE10246), we confirmed that T1/ST2 was primarily expressed on MCs. Consistent with our results in vitro, IgE and IgE + antigen treatments resulted in lower T1/ST2 expression compared with the normal condition (Fig. 5h). Recovery after degranulation of MCs promoted T1/ST2 expression (Fig. 5i). Therefore, we hypothesized that some MCs in the inflamed colon went through the degranulation-restore process to promote T1/ST2 expression.

### MC-dependent IL-33/ST2 signals promote mucosal healing

**Fig. 6:**
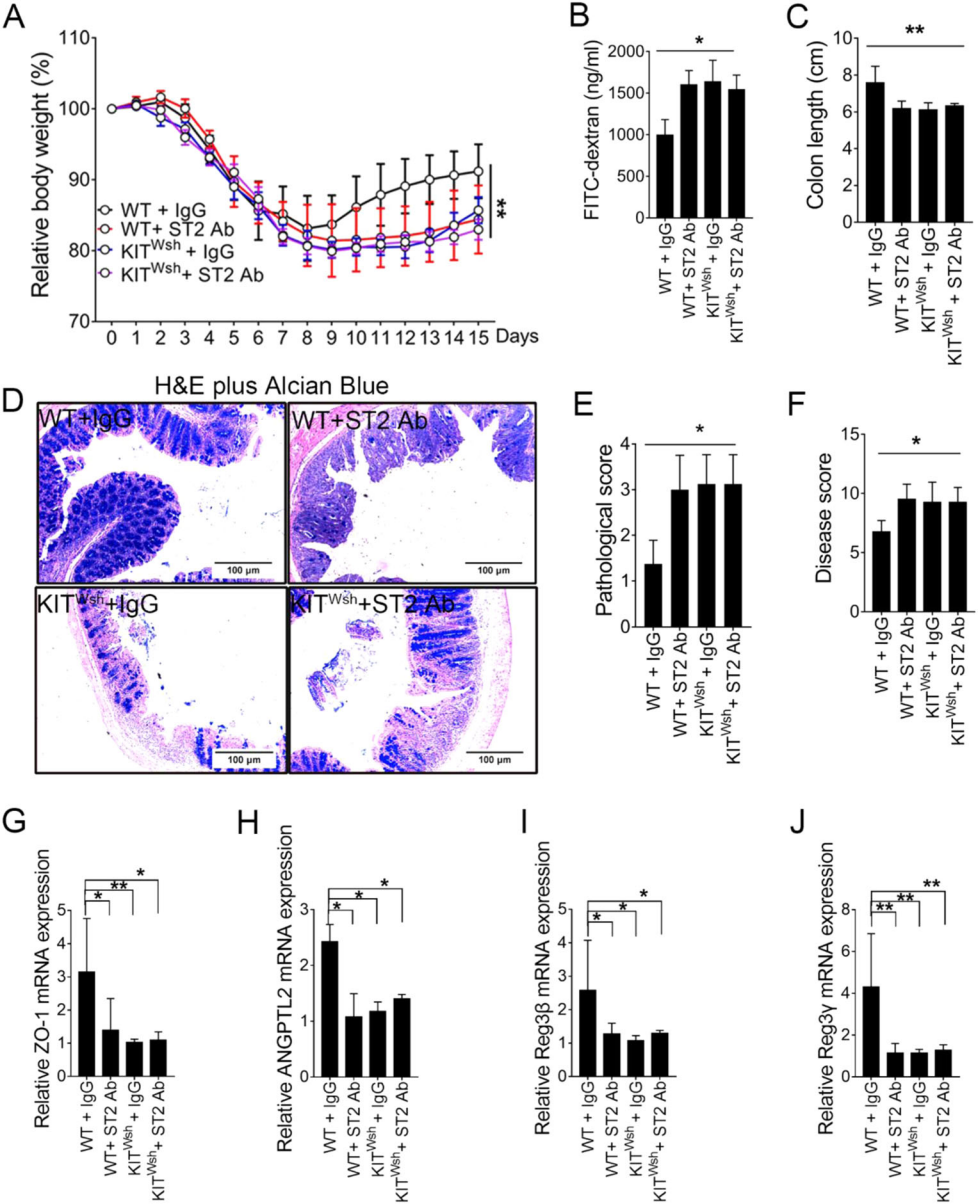
MC-dependent IL-33/ST2 signaling pathway is required for recovery from colitis. **a** Percentage difference in mass from day 0 for WT + IgG, WT + anti-ST2 Ab, Kit ^Wsh^  +  IgG and Kit ^Wsh^  +  ST2 Ab groups (6 mice/group). **b** Serum FITC-dextran quantified as a measure of intestinal permeability. **c** Measurement and comparison of colon length in remission phase. **d** Representative H&E plus alcian blue staining images of transverse colon (original magnification ×400). **e** Pathological grading of colitis. **f** Disease score from a comprehensive evaluation as indicated. Relative mRNA for **g** ZO-1, **h** ANGPTL2, **i** Reg3β, and **j** Reg3γ from real-time-quantification PCR. Data were pooled from three repeated experiments. Data are mean ± SD. Student *t-*test, *P < 0.05; **P < 0.01

To formally assess the role of MC-dependent IL-33/ST2 signals in intestinal homeostasis, we explored if absence of the IL-33-mediated signaling pathway promoted colitis recovery in MC-deficient KIT ^Wsh^ or WT mouse models. All DSS-treated mice had significant progressive weight loss over the acute phase (Fig. 6A). Body weight loss with WT mice treated with blocking anti-ST2 antibody was comparable to KIT ^Wsh^ mice in the acute and recovery phases of colitis. This result was confirmed by FITC-dextran assays, colon length, histological analyses, and disease scores (Fig. 6b–f). No significant difference in tissue recovery was seen in KIT ^Wsh^ mice treated with isotype or anti-ST2 antibodies, suggesting MCs were essential for a redundant IL-33 signal to promote mucosal healing. We also demonstrated that an IL-33/ST2 signaling pathway in MCs was required for expression of ZO-1, ANPTL2, Reg3β, and Reg3γ, which were associated with signal transduction and immune regulation (Fig. 6g–j).

### MC-dependent IL-33/ST2 signal orchestrates an immune network favorable to mucosal healing

**Fig. 7:**
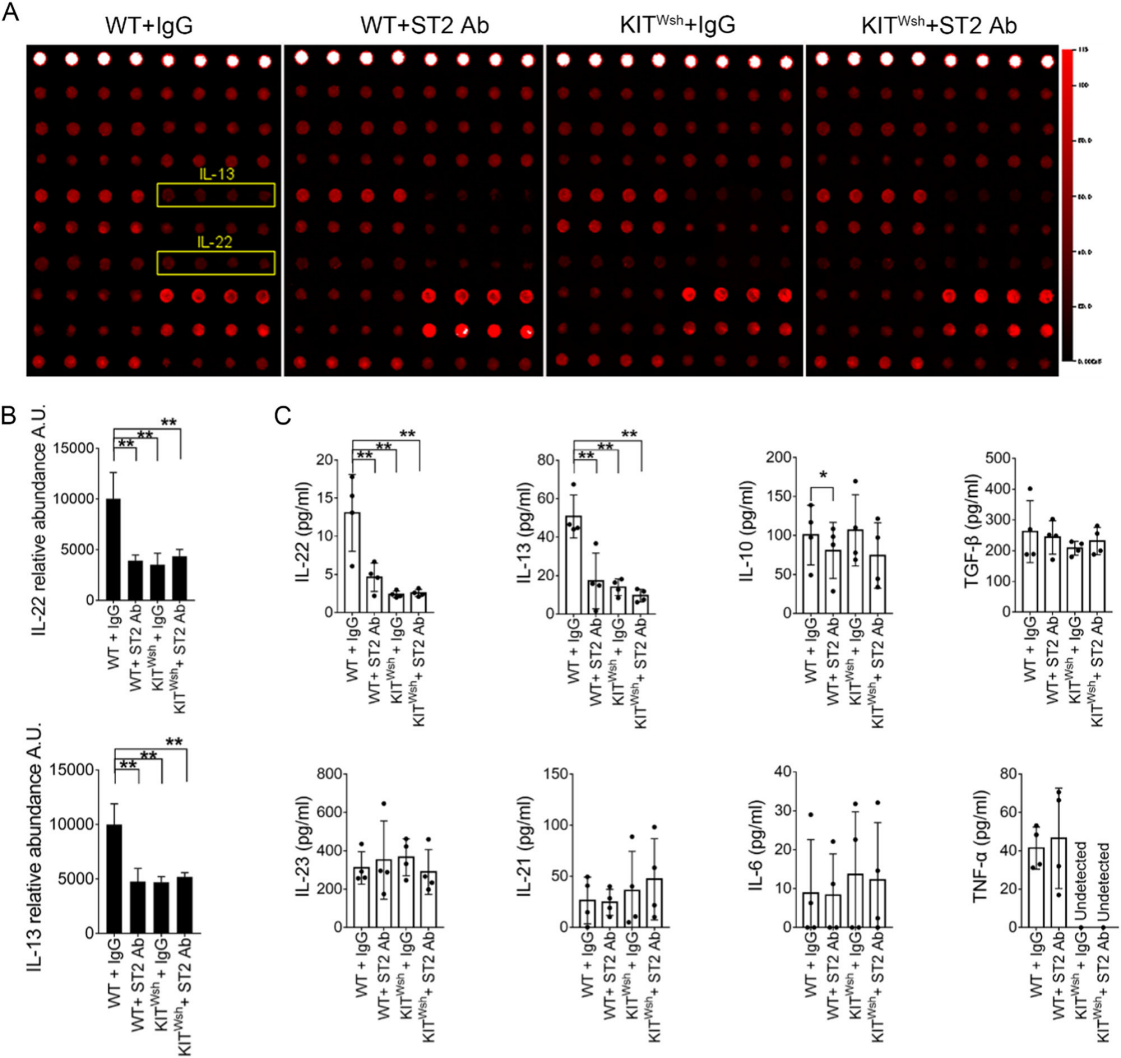
MC-dependent IL-33/ST2 signal pathway promotes IL-22 and IL-13 production in the remission phase. **a** Supernatants from colon tissue in the remission phase analyzed by protein arrays detecting 18 different cytokine factors. Examples of developed membranes with indications of factors of interest associated with IL-33/ST2 signaling function in MCs. **b** Relative abundance of IL-22 and IL-13. **c** Concentration of IL-22, IL-13, IL-10, TGF-β, IL-23, IL-21, IL-6, and TNF-α in supernatants of colon tissue by ELISA (*n* = 4). Data are mean ± SD. Student *t-*test, *P < 0.05; **P < 0.01

To identify potential tissue-specific cytokines of immune cells, we compared protein expression profiles of colon tissue. We identified IL-13 and IL-22 as important cytokines from colon tissue (Fig. 7a, b). To further assess and confirm the effect of MC-dependent IL33/ST2 signaling on the colon tissue immune network during the remission phase, we used enzyme-linked immunosorbent assay (ELISA) to quantified the expression of potentially important cytokines from T cells. Only decreased expression of IL-13 and IL-22 was largely and tightly restricted to blocking the MC-dependent IL33/ST2 pathway (Fig. 7c).

### IL-33 signal facilitates MCs survival and function in vitro

**Fig. 8:**
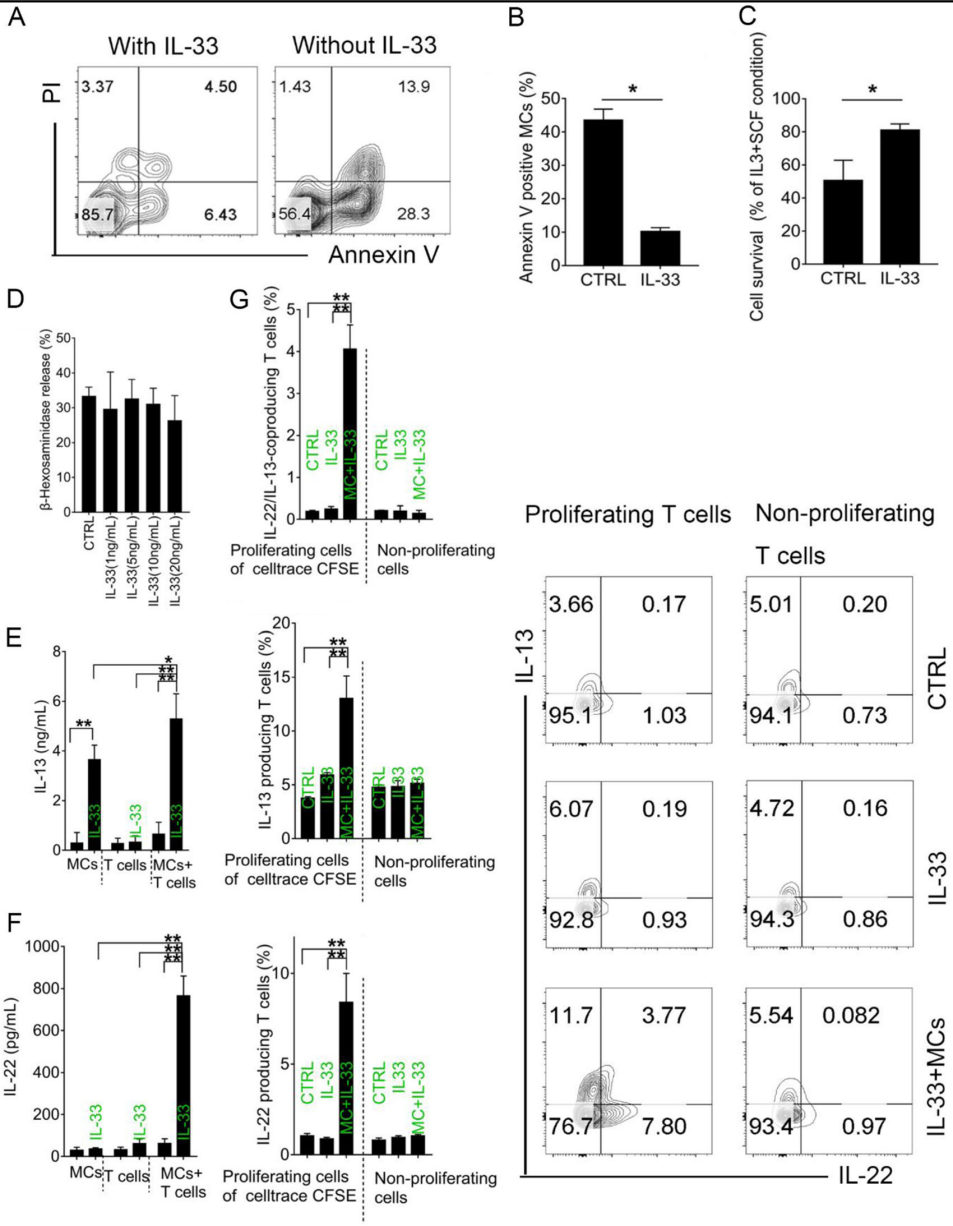
IL-33 signal promotes MCs survival and function in vitro. **a** Representative plots of apoptosis assays of MCs treated with or without IL33 in the absence of SCF and IL-3. **b** Mean apoptosis percentage. Data were pooled from three repeated experiments. **c** Proliferation rate of MCs treated with or without IL-33 in the absence of SCF and IL-3, divided by results from regulate culture conditions (medium with IL-3 and SCF). **d** MC degranulation assessed by β-hexosaminidase release. Concentrations of **e** IL-13 and **f** IL-22 in supernatants of MCs, T cells or MCs co-cultures with T cells, by ELISA. **g** Percentage of IL-22/IL-13-coproducing T cells, IL-13-producing T cells, and IL-22-producing T cells in proliferating T cells by cell trace CFSE or nonproliferating cells assessed by flow cytometry. Plots used data from three repeated experiments. Data are mean ± SD. Student *t-*test, *P < 0.05; **P < 0.01

We assessed the impact of IL-33 on MCs in vitro. In line with previous findings, we showed that IL-33 attenuated apoptosis of murine BMMCs, promoting their survival (Fig. 8a–c). MC degranulation was not promoted by IL-33 alone (Fig. 8d). Given that T cells are prominent in the colon, we postulated that IL-33/ST2 signaling in MCs may modulate in vitro-induced T cell differentiation and function. To test this, we sort-purified naïve CD4+T cells from WT mice and cocultured them with or without BMMCs. Consistent with in vivo results, both IL-13 and IL-22 secretion were promoted by IL-33 in the co-culture system. IL-13 was secreted by MCs alone, confirming that MCs were an important source of Th2 cytokines (Fig. 8e, f). The presence of IL-33 in MCs cultures significantly promoted IL-13-producing and IL-22-producing T cell percentage in the proliferation phase, suggesting that IL-33 preferentially depended on intermediary MCs to regulate T cell differentiation and function (Fig. 8g).

## Discussion

Animal and human clinical studies that have implicated the participation of MCs in IBD^11–13,31,32^ suggest that the contribution of these cells includes regulating epithelium permeability, immune signal transmittance, maintenance and resolution of inflammatory responses, and subsequent tissue remodeling.^33^ Our data provide evidence for a tissue-protective function for MCs in the intestinal tract and implicate the IL-33/ST2 signaling pathway as a critical mechanism by which MCs mediate the production of IL-13 and IL-22 to orchestrate the immune network to limit inflammation and promote epithelial repair. This study identified a critical feedback loop in which cytokine signals from damaged epithelia mediate innate immune cell responses and promote expression of a spectrum of cytokines essential for restoration of epithelial barrier function and tissue homeostasis. Strategies designed to regulate this feedback loop may provide novel therapeutic agents for IBD management.

Accumulating evidence indicates that the function of MCs in IBD is more complicated than originally thought, for example, the composition of MCs and how their phenotypes can be altered under various conditions. However, this evidence also raises the opportunity to explore information about MCs and their associated signaling pathways in IBD. In this study, we confirmed the dynamic changes of MC infiltration in inflamed colons and MC functions in repairing DSS-induced colon damage. Consistent with previous studies,^13^ the increase in MC number in the lamina propria of WT mice after withdrawal parallels the delayed recovery of weight loss occurring in KIT ^Wsh^ mice. Although we have no evidence that MCs have a special function in the activation phase of colitis, signaling such as SCF released from damaged tissue in this phase was essential for facilitating MC development and survival in the subsequent remission phase. Our findings highlighted MC functions in colonic epithelial regeneration and intestinal homeostasis suggested by indicators for detecting colitis and by Ki-67 immunostaining. The increase of MC infiltration near the epithelium resulted in a favorable microenvironment that expressed more beneficial proteins including ZO-1, FGF-2, ANGPTL2, REG3γ, and REG3β, which are involved in signal transduction, cell growth, tissue repair, and homeostasis maintenance. Thus, we have added new insights about a novel innate immune component to the pathway that involves these factors and suggest that intestinal tissue protection requires a progressive MC response that is initiated by rapid damage signals.^33^

IL-1 family member IL-33 is constitutively expressed in epithelial cells at barrier sites where it functions as an endogenous danger signal or alarmin following tissue damage.^34,35^ Although high levels of IL-33 are found in inflamed intestinal segments, how IL-33 functions in colitis has remained a formidable challenge due to diverse conclusions from previous studies.^19,20,30,36–38^ Our data suggested that delayed resolution of inflammation in the KIT ^Wsh^ mice was accompanied with a failure to reduce IL-33. MC granules contain a wide range of proteases and mMCP-4 degrades several alarmins, including IL-33, both in vitro and in vivo.^39^ Based on our findings and previous studies^40–43^ implicating MC with IL-33/ST2, we investigated the relationship of MC activity and ST2 expression during colitis development. Our data identified that MCs acted as the primary cell type that expressed ST2 among the CD45 + CD11b− lamina propria mononuclear cells (LPMCs) during colitis development and among immune cells and important functional cells. In addition, recovery after MC degranulation promoted ST2 expression. Taken together, these results suggested the development and function of MCs was involved in the IL-33/ST2 signaling pathway.

From an immune perspective, MCs appear to be cellular sensors and effectors for IL-33, in keeping with their functional relationship with other systems, where they lead to different biological endpoints.^10,21,24,44–48^ However, further assessing if IL-33 is required for MC response and function during colitis is still necessary. We identified MC-dependent IL-33/ST2 signaling as one pathway for orchestrating intestinal tissue protection, consistent with IL-33 function in previous studies. However, we did not observe significant differences in colitis degree between groups with or without ST2 blocking in KIT ^Wsh^ mice. IL-33 is a potent in vivo stimulus that activates different resident immune cell populations in multiple inflammatory settings.^49^ We remain confident that IL-33 has a key function in activating other cell populations but in colitis, this process in largely thought to act via MC-dependent signaling. Supporting our view is the decreased expression of ZO-1, ANGPTL2, Reg3β, and Reg3γ in ST2-blocked groups. Consistent with our findings, a study suggests that the IL-33-MC axis suppresses tissue inflammation with an immunosuppressive negative-feedback role in airways,^50^ suggesting that downstream signals from MCs involving IL-33 orchestrate a specific immune microenvironment.

The cellular and molecular mechanisms acting downstream of IL-33 to regulate disease are poorly defined. To some extent, we demonstrated that IL-33 signaling activated MCs to form a tissue microenvironment favorable to tissue repair. Given the known influence of IL-33 on signals from T cells,^35^ exploring if intestinal tissue protection requires a cooperative response from both MCs and T cells to expand downstream signals to orchestrate a special immune network to facilitate tissue repair is worthwhile. We identified IL-22 and IL-13 as significantly downregulated cytokines preferentially involved in blocking MC-dependent IL-33/ST2 signaling using protein array and ELISA assays. IL-33 is a well-known regulator of mucin responses in multiple inflammatory settings, but our colitis model largely found IL-13- and IL-22-dependent signaling. MCs are long-lived cells that accumulate in chronically inflamed tissues,^51^ consistent with the long process of colon epithelium repair during colitis. Despite the gradual decline in IL-33 levels with increasing repair of intestinal mucosal damage, IL-33 also contributes to MC survival and function and possibly until damaged colon tissue recovers the normal structure. In line with the results of a previous study, our data revealed a cell-intrinsic role for IL-33 signal in the regulation of MC apoptosis.^52^ Considering that we did not examine directly whether MC-derived ST2 plays a role, for instance, using mice that are deficient of ST2 only in the MC population, the potential role of other possible confounding factors cannot be completely ruled out during the both the acute and recovery phases of colitis. Nevertheless, MCs are tissue-based stationary effector cells that present a first-line defense against challenges and are closely related to the inflammation process.^51^ For these reasons, MCs are more prominent in the remission phase.

IL-13 was largely released from MCs treated with IL-33, with more production in an MC-T cell co-culture system. Most biological effects of IL-13, similar to those of IL-4, are linked to a single transcription factor, signal transducer and activator of transcription 6 (STAT6).^53^ IL-13 is responsible for promoting the survival and migration of epithelial cells,^54^ consistent with our findings. Similarly, IL-22R is specifically expressed in epithelial cells and IL-22 acts directly on epithelium, which is thought to be beneficial for the intestinal epithelial barrier by enhancing mucus production and promoting the proliferation and migration of epithelial cells.^55,56^ Therefore, a powerful proliferative stimulus linking IL-33, MCs, IL-13, and IL-22 to neighboring epithelia in vivo maybe sufficient for tissue repair with increased cytokine expression including ANGPTL2, Reg3β, and Reg3γ.

In summary, our data indicated that MCs, as important tissue-resident effector cells with a long life in inflamed colons, were essential intermediaries in regulating IL-33/ST2 signaling to orchestrate an immune network favorable to mucosal healing. Furthermore, production of IL-13 and IL-22 mediated by an MC-dependent IL-33/ST2 pathway, as a previously unrecognized multiple mechanism, was responsible for the beneficial effects of tissue homeostasis and immune regulation. However, an open area of investigation remains if human intestinal MCs have a consistent function as murine MCs for resolution of colon inflammation, and long term, if the repair capacity of MCs is also adjusted by genetic alterations in tumor-associated modification.

## Materials and methods

### Animals and treatments

Mice were maintained under pathogen-free conditions and housed in filter-top cages. All animal experiments were conducted with the approval of the Local Ethics Committee responsible for regulating animal research in Tongji University. Animal care was strictly according to the guidelines for the care and use of laboratory animals published by the US National Institutes of Health (NIH Publication No. 85-23, revised 1996). Wild-type (WT) C57BL/6 mice were from Shanghai Laboratory Animal Co Ltd. (Shanghai, China), and MC-deficient mice C57BL/6-Kit^W-sh/W-sh^ (Kit ^Wsh^) were from Jackson Laboratories (Bar Harbor, ME, USA). Reconstitution of KIT ^Wsh^ mice with murine bone marrow-derived MCs (BMMCs), termed the “mast cell knock-in” approach, was performed by intraperitoneal (i.p.) injection of 5 × 10^6^ cells per mouse. Wait 5 weeks after i.p. engraftment before performing in vivo experiments. DSS-induced colitis models were established using a method described previously.^57^ WT and Kit ^Wsh^ mice were give 2.5% DSS (molecular mass, 36,000–50,000; MP Biomedicals) in drinking water for 7 days continuously, then the recovery phase was evaluated followed with regular water. Characteristics of acute colitis were observed daily, including diarrhea, rectal bleeding, body weight, and survival. Disease severity was scored as in a previous study.^58^ ST2 neutralization was by i.p. administration of 20 μg anti-ST2 blocking antibody or rat anti-mouse IgG2b antibodies as the isotype control (R&D Systems).

### Cell preparation

BMMCs were differentiated and cultured. Bone marrow cells were isolated from femurs of C57BL/6 mice, differentiated in complete RPMI (Gibco), and supplemented with 10% FBS (Gibco), 100 U/mL penicillin/streptomycin (Gibco), 10 ng/mL recombinant murine IL-3 (R&D Systems), and 10 ng/mL recombinant murine SCF (R&D Systems) at 37 °C under 5% CO_2_. After 7 days, nonadherent cells were carefully removed and replaced with fresh culture medium to increase the purity of the MCs. This step was repeated every 7 days until adherent cells disappeared (after 5–6 passages). After 5 weeks of culture, BMMC purity was evaluated as the percentage of FcεRIα and c-kit (CD117) cells. BMMCs were used at a purity more than 90%. CD4^+^ T cells were enriched using an isolation kit (Miltenyi Biotech) according to the manufacturer’s instructions. To analyze immune cells infiltrating the colon, collagenase IV (0.05% w/v; Roche) was used to digest the tiny colon tissue (0.1–0.5 cm) for 1 h. Single-cell suspensions were collected and further purified via density gradient centrifugation with 40% (v/v) and 70% (v/v) Percoll-RPMI. LPMCs were collected from the interface and suspended in RPMI medium.^59^

### Histopathology

Colons isolated from euthanized animals were embedded in paraffin and 5-μm sections were deparaffinized in xylene and hydrated through a graded series of alcohol to water. For toluidine blue staining (Sigma) to assess MCs in the colon, slides were rinsed with PBS for 10 min and stained with 0.5% w/v toluidine blue (Sigma) in 0.5 N HCl (Baker, Philipsburg) for 30 min. Staining with H&E (Sigma) or H&E plus Alcian Blue was under standard conditions. Slides were dehydrated and mounted in Richard-Allan Scientific Cytoseal XYL (Invitrogen). Histology was scored blindly by lab technicians from the Department of Pathology using previously described criteria: 0, no signs of inflammation; 1, very low level of inflammation; 2, low level of leukocyte infiltration; 3, high level of leukocyte infiltration, high vascular density, thickening of the colon wall; and 4, transmural infiltration, loss of globet cells, high vascular density, thickening of the colon wall. For immunofluorescence or immunohistochemistry, antigen retrieval was performed by microwave irradiation in citrate buffer for 20 min and cooling to room temperature. Sections were incubated with 3% H_2_O_2_ in distilled water for 15 min to quench endogenous peroxidase activity. After rinsing three times with PBS, sections were incubated with Ki-67 IgG antibody (2.5 μg/mL; 12202, CST) and IL-33 IgG antibody (2 μg/mL, ab229698; Abcam) overnight at 4 °C, and washed in PBS. Sections were incubated with secondary antibody for 1 h at room temperature. Negative controls were processed in the same manner but without primary antibody.

### FITC-labeled dextran intestinal permeability assays

Intestinal permeability was examined using a FITC-labeled dextran method. Mice were gavaged with 60 mg/100 g FITC dextran (MW 4000 at 80 mg/mL; Sigma) 4 h before sacrifice. Sera were obtained to detect FITC-dextran levels using fluorescence microplates and an excitation maximum of 490 nm and emission maximum of 520 nm (Fluorimeter Pharos FX; BioRad). Standard curves were obtained by diluting serial concentrations of FITC-dextran in mouse serum.

### Real-time quantitative PCR

We extracted RNA using the TRIzol method (Invitrogen) and purified it using RNeasy columns (Qiagen). Real-time PCR was with Light Cycler RNA Master SYBR Green kits (Roche) in a LightCycler instrument (Roche) using primers specific for detected genes (Table S1). On completion of amplification, DNA melting curve analysis was carried out to confirm the presence of a single amplicon. GAPDH was the internal reference gene for normalizing transcript levels. Relative mRNA levels (2−ΔΔCt) were determined by comparing PCR cycle thresholds (Ct) for the gene of interest and GAPDH (ΔCt) between WT and KIT ^Wsh^ groups.

### Antibody staining and flow cytometry

Cells were preincubated 10 min with anti-CD16/32 FcγII/III (BD Bioscience) to prevent nonspecific binding, washed and stained for surface markers, fixed in Fix/Perm buffer (BD Bioscience), and permeabilized in permeabilization buffer (BD Bioscience) for 1 h with antibodies. For intracellular staining, GolgiStop (BD Bioscience) was added 6 h before Fc block, fixation, and permeabilization (BD Bioscience). Primary antibodies for flow cytometry were: PerCP/Cy5.5-conjugated anti-CD45 (BD Biosciences), APC-conjugated anti-CD11b (BD Bioscience), PE-conjugated anti-CD117/c-Kit (BioLegend), FITC-conjugated anti-FcεRIα (BioLegend), PE/Cy7-conjugated anti-T1/ST2 (IL-33R; eBioscience), APC-conjugated anti-IL-22 (eBioscience), and PE/Cy7-conjugated anti-IL-13 (eBioscience). Acquisition was with a BD FACSCanto instrument. Data analyses used FlowJo software (Tree Star).

### Protein arrays and ELISA assays

Colon tissue was homogenized in 1 mL PBS and centrifuged (13,000 rpm for 20 min at 4 °C). Supernatant was filtered (0.22 μm), and protein concentration was determined by Bradford assay. The presence of cytokines was analyzed in supernatant fractions with mouse antibody arrays Q1 (ABIN625794; RayBiotech) following the manufacturer’s instructions. ELISA kits were used to quantify IL-22, IL-13, IL-10, TGF-β, IL-23, IL-21, IL-6, and TNF-α (RayBiotech).

### MC degranulation

Blood samples were drawn into serum separation tubes and serum aliquots stored at −80 °C until analysis. Histamine concentrations were assessed using EIA histamine kits according to the manufacturer’s instructions (Beckman Coulter). Serum was acylated and added to histamine-coated plates with conjugate and incubated overnight at 4 °C. Wells were washed and substrate added for 30 min at room temperature while shaking. Reactions were stopped and plates read at 405 nm. In vitro, BMMCs were incubated overnight at 37 °C with 1 µg/mL IgE anti-DNP in 1 mL culture medium and stimulated for 1 h at 37 °C with 10 ng/mL DNP-BSA antigen in 200 µL Hepes-Tyrode’s buffer, pH 7.4. Culture supernatants from cells were collected and assayed for β-hexosaminidase.

After solubilization with 0.5% (vol/vol) Triton X-100 in Tyrode’s buffer, enzymatic activities of β-hexosaminidase in supernatants and cell pellets were measured with *p*-nitrophenyl *N*-acetyl-b-d-glucosaminide in 0.1 M sodium citrate (pH 4.5) for 60 min at 37 °C. Reactions were stopped by addition of 0.2 M glycine (pH 10.7). Release of 4-*p*-nitrophenol product was detected by absorbance at 405 nm. Extent of degranulation was represented as 4-*p*-nitrophenol absorbance in supernatants as a percentage of sum of absorbance in supernatants and in cell pellets solubilized in detergent.

### Determination of MC survival

MCs were treated with or without IL-33 after withdrawing cytokines IL-3 and SCF, which are essential for MC survival. For MC apoptosis analysis, cells were stained with annexin V (eBioscience) at room temperature in the dark for 15 min, followed by the addition of propidium iodide. After staining, the percentage of apoptotic cells among 10,000 cells was analyzed using flow cytometry. The Q2 region represents late apoptotic cells, and the Q4 region represents early apoptotic cells. To evaluate MC survival and proliferation, a cell counting Kit-8 (CCK-8; Dojindo) was used according to the manufacturer’s instructions.

### Statistical analysis

Results were expressed as mean values ± SD. Differences among experimental groups were evaluated via Student’s *t*-test or ANOVA when appropriate, and significance of differences between groups assessed via Newman–Keuls post hoc test. Analysis used statistical software package (GraphPad-Prism 7; GraphPad, La Jolla, CA, USA) and significance was defined as *P* < 0.05.

## Electronic supplementary material

Supplementary figures

Supplementary figure legends
