# Supplementary figures and images for: Mast cells are essential intermediaries in regulating IL-33/ST2 signaling for an immune network favorable to mucosal healing in experimentally inflamed colons
Source: Cell Death Dis. 2018 Dec 5;9(12):1173. doi: 10.1038/s41419-018-1223-4 (PMC6281667; doi:10.1038/s41419-018-1223-4)

Figure 1S.

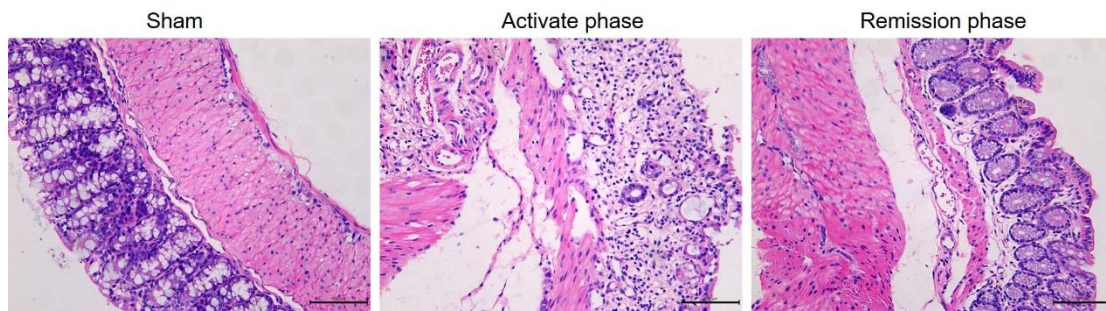

Figure 2S

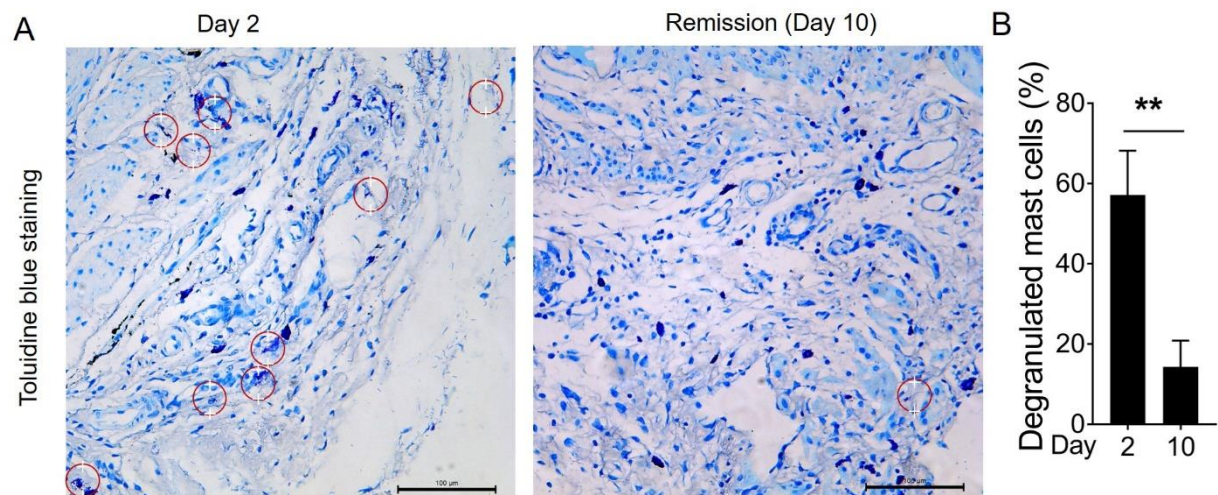

Supplement: Supplementary file 1 — Supplementary figures [file 41419_2018_1223_MOESM1_ESM.pdf]
