# Supplementary material for: Mast cells are essential intermediaries in regulating IL-33/ST2 signaling for an immune network favorable to mucosal healing in experimentally inflamed colons
Source: Cell Death Dis. 2018 Dec 5;9(12):1173. doi: 10.1038/s41419-018-1223-4 (PMC6281667; doi:10.1038/s41419-018-1223-4)
Supplement: Supplementary file 2 — Supplementary figure legends [file 41419_2018_1223_MOESM2_ESM.docx]

**Figure s1. Colon pathology (H&E staining) of mice subjected to sham treatment or 2.5% DSS insult.**

**Figure s2. The analysis of degranulated MCs in the colon.**

(A). Degranulation detection of colon MCs by using toluidine blue staining at day 2 and 10 during colitis. (B) Analysis of the percentage of degranulated MCs. Data are mean ± SD. Student *t-*test, ** *P* < 0.01.

| **Gene Name** | **Sequence (5' -> 3')**  Forward Primer  Reverse Primer |
| --- | --- |
| GAPDH | AGGTCGGTGTGAACGGATTTG  TGTAGACCATGTAGTTGAGGTCA |
| SCF | GAATCTCCGAAGAGGCCAGAA  GCTGCAACAGGGGGTAACAT |
| c-KIT | CTCCCCCAACAGTGTATTCAC  TAGCCCGAAATCGCAAATCTT |
| mMCP7 | GCCAATGACACCTACTGGATG  GCTTACGGAGCTGTACTCTGA |
| ZO-1 | GCCGCTAAGAGCACAGCAA GCCCTCCTTTTAACACATCAGA |
| LRH-1 | TGAGGAACAACTCCGGGAAAA CAGACACTTTATCGCCACACA |
| NURR1 | GTGTTCAGGCGCAGTATGG  TGTATTCTCCCGAAGAGTGGTAA |
| FGF-2 | GCGACCCACACGTCAAACTA TCCCTTGATAGACACAACTCCTC |
| REG3γ | ATGCTTCCCCGTATAACCATCA GGCCATATCTGCATCATACCAG |
| REG3β | CCCTCCGCACGCATTAGTT CAGGCCAGTTCTGCATCAAA |
| RIPK1 | GACAGACCTAGACAGCGGAG CCAGTAGCTTCACCACTCGAC |
| ANGPTL2 | CCACCTCGGGTCTACCAAC  CTTGCAGGCAGTCTCTCCAT |
| IRF3 | GAGAGCCGAACGAGGTTCAG CTTCCAGGTTGACACGTCCG |
| LRIG1 | TTGAGGACTTGACGAATCTGC CTTGTTGTGCTGCAAAAAGAGAG |
| P16 | CGCAGGTTCTTGGTCACTGT  TGTTCACGAAAGCCAGAGCG |
| C-FLIP | GCTCCAGAATGGGCGAAGTAA  ACGGATGTGCGGAGGTAAAAA |
| IL-33 | TCCAACTCCAAGATTTCCCCG  CATGCAGTAGACATGGCAGAA |
| T1/ST-2 | TGACACCTTACAAAACCCGGA  AGGTCTCTCCCATAAATGCACA |

**Table s1. Primers for Quantitative RT-PCR**
